# Understanding the Redox Biology of Selenium in the Search of Targeted Cancer Therapies

**DOI:** 10.3390/antiox9050420

**Published:** 2020-05-13

**Authors:** Jeffrey M. Stolwijk, Rohan Garje, Jessica C. Sieren, Garry R. Buettner, Yousef Zakharia

**Affiliations:** 1 Interdisciplinary Graduate Program in Human Toxicology, The University of Iowa, Iowa City, IA 52242, USA; garry-buettner@uiowa.edu; 2 Department of Internal Medicine, Division of Medical Oncology and Hematology, The University of Iowa Hospital and Clinics—Holden Comprehensive Cancer Center, Iowa City, IA 52242, USA; rohan-garje@uiowa.edu; 3 Departments of Radiology and Biomedical Engineering, The University of Iowa, Iowa City, IA 52242, USA; jessica-sieren@uiowa.edu; 4 Free Radical and Radiation Biology Program, Department of Radiation Oncology, The University of Iowa, Iowa City, IA 52242, USA

**Keywords:** selenium, selenomethionine, selenoproteins, cancer, glutathione peroxidases, thioredoxin reductases

## Abstract

Selenium (Se) is an essential trace nutrient required for optimal human health. It has long been suggested that selenium has anti-cancer properties. However, clinical trials have shown inconclusive results on the potential of Se to prevent cancer. The suggested role of Se in the prevention of cancer is centered around its role as an antioxidant. Recently, the potential of selenium as a drug rather than a supplement has been uncovered. Selenium compounds can generate reactive oxygen species that could enhance the treatment of cancer. Transformed cells have high oxidative distress. As normal cells have a greater capacity to meet oxidative challenges than tumor cells, increasing the flux of oxidants with high dose selenium treatment could result in cancer-specific cell killing. If the availability of Se is limited, supplementation of Se can increase the expression and activities of Se-dependent proteins and enzymes. In cell culture, selenium deficiency is often overlooked. We review the importance of achieving normal selenium biology and how Se deficiency can lead to adverse effects. We examine the vital role of selenium in the prevention and treatment of cancer. Finally, we examine the properties of Se-compounds to better understand how each can be used to address different research questions.

## 1. Introduction

Selenium (Se) is a trace element, essential for optimal health. In the mid 20th century selenium was found to be protective in liver tissue [1,2,3]. It was not until the 1970s that Flohé et al. discovered the presence of selenium at the active site of an enzyme, glutathione peroxidase 1 (GPx1) [4]. Later it was determined that selenium was at the active site in the form of a proteinogenic amino acid, selenocysteine (Sec) and that this Se was essential for the activity of the enzyme [5]. Since this first discovery, 25 selenoproteins have been identified that contain one or more Sec.

The RDA (recommended daily allowance) of Se for both men and women is 55 μg (0.7 μmol)/day [6]. In the diet the major forms of selenium are highly bioavailable. The Tolerable Upper Intake Level (UL) for adults is 400 μg (5.1 μmol)/day, based on selenosis as the adverse effect. Selenium deficiency compromises the selenium-dependent antioxidant system. Supplementation of selenium can maximize the expression and activities of selenium-dependent proteins and enzymes [7,8,9].

However, at levels above the UL, Se compounds are commonly thought to be associated with toxicity. Still, there have been many human clinical trials that show beneficial properties with very high, supranutritional levels of selenium, without significant toxicity [10,11]. At these high levels, selenoenzyme expression is not increased above levels associated with the RDA in humans [7,8,9]. Therefore, other mechanisms must contribute to the possible benefits of high-dose Se in the treatment of cancer. Metabolites of selenium compounds can contribute to hydrogen peroxide generation, perhaps via formation of superoxide, and cysteine oxidation and eventually DNA damage [12,13,14]. There appears to be potential for the use of these prooxidant traits of selenium in cancer therapies.

Cell culture models (i.e., in vitro) are used by researchers worldwide in the field of cancer research. The use of these models is a key first step before proceeding to preclinical animal studies. In 1985, Speier et al. suggested that cell culture media were deficient in Se [15]. They observed that this deficiency blunted the activity of GPx1 in HL-60 cells. Adding selenium to the cell culture medium overcame this deficiency as the activity of GPx1 was substantially increased. This work and the work of others indicated that Se is invariably deficient in cell culture media [16,17,18]. This observation is widely underappreciated.

This review focuses on the role of selenium in the prevention and treatment of cancer. We discuss the potential consequences of having insufficient selenium in in vitro models used in cancer research. We also summarize knowledge on the function of selenium when used in cancer treatment.

## 2. Selenoproteins in Redox Biology and Cancer

It is well accepted that selenoproteins are maximally expressed under sufficient nutritional selenium levels. Selenoproteins are a class of proteins, with or without enzymatic properties, that contain one or more selenium atoms usually in the form of a selenocysteine (Sec) moiety (Table 1). The family of Se-proteins is widely distributed throughout organs and tissues, where both ubiquitous and tissue-specific Se-proteins are expressed [19]. Selenoproteins have a wide variety of physiological roles, e.g., protection against oxidative stress, selenium transport, regulation of thyroid hormones, and synthesis of DNA. In addition, subcellular localization gives individual family members specificity for substrates and biological roles. Glutathione peroxidases (GPx), for example, have specific localized family members; GPx1 is predominantly abundant in the cytosol while GPx4 is mainly associated with the lipid bilayers of the cell membranes. While some functions may seem similar, e.g., removal of hydroperoxides, location is important for the substrate specificity of the enzyme. For GPx1 organic hydroperoxides must be solubilized to be a substrate, e.g., phospholipid hydroperoxides must be cleaved from a membrane by phospholipase A_2_ yielding a fatty acid, as a carboxylate, that is soluble in the cytosol [20]; whereas GPx4 acts on phospholipid hydroperoxides that are membrane-bound, i.e., localized within the phospholipid bilayer [21,22].

### 2.1. Glutathione Peroxidases

The glutathione peroxidase family was the first selenium-containing enzyme family identified [4,5,23], making it one of the best characterized set of selenoproteins. GPx family members 1–4, and 6 contain Sec at the active site of the enzyme. Other members rely on cysteine residues for activity. GPxs are generally thought to bring about the two-electron reduction of hydrogen peroxide or organic hydroperoxides (Equation (1)).

(1) $$\mathrm{ROOH}\;+\;2\mathrm{GSH}\;\overset{GP_{x}}{\to }\;\mathrm{ROOH}\;+\;H_{2}O\;+\;\mathrm{GSSG},\;\mathrm{where}\;R\;=\;H\;\mathrm{is}\;\mathrm{allowed}$$

Selenium is found below sulfur in the periodic table of elements; thus, they share some chemical properties. The main difference is the bulky valence electron shell of selenium compared to sulfur. When integrated as selenocysteine into GPxs, this facilitates the reduction of hydrogen peroxide, and organic hydroperoxides, including lipid hydroperoxides. Upon reduction of these potential toxicants, the ionized selenol (R-Se^-^) at the active site of GPx is oxidized to selenenic acid (R-SeOH). Two glutathione molecules provide the two electrons to reduce the active site selenenic acid back to selenol [4,5,24].

The principal physiological role of GPx is to protect cells and tissues from oxidative fluxes of hydroperoxides. These fluxes occur through normal biological processes. For example, mitochondrial respiration generates superoxide that can be dismuted by superoxide dismutases (SOD) to hydrogen peroxide. Intra and extracellular superoxide is formed by NADPH oxidases, nitric oxide synthase, cytochrome p-450s, and xanthine oxidase [25]. Increased fluxes of superoxide can result in an increased steady-state level of hydrogen peroxide. If left unchecked, this can lead to the initiation of lipid peroxidation, oxidation of proteins, and oxidative DNA damage [26].

#### 2.1.1. GPx1

GPx1, also known as cytosolic GPx as well as classic GPx, is one of the most abundant and ubiquitously expressed selenoproteins [27,28]. Its expression is influenced by oxidative eustress and oxidative distress as well as fluctuations in the availability of Se, making it one of the most sensitive selenoproteins [29]. Several studies have demonstrated an association between GPx1 and cancer when expression is repressed or reduced [24,26,30,31,32]. In MCF-7 breast carcinoma or mouse fibroblasts, GPx1 protects cells from DNA damage induced by UV light [33]. This effect was reversed when selenium was deficient in the cell culture media, consistent with a lower activity of GPx1. It has also been suggested that GPx1 can prevent the initiation of carcinogenesis through its counteractive role during the inflammation response of neutrophils [34]. Furthermore, estrogen can likely upregulate GPx1 upon oxidation of the NFκB cascade [35,36]. Hydroperoxides, a substrate for GPx1, can stabilize p53. This prevents tumor cells from proliferating and induces tumor-specific apoptosis [37]. Moreover, the promotor of *Gpx1* contains a p53 binding site indicating that p53 can activate the transcription of GPx1. It appears that GPx1 mainly contributes to the prevention of oxidant-mediated toxicity that could result in the initiation of carcinogenesis. However, the antioxidant characteristic of GPx1 can prevent oxidant-induced apoptosis in transformed cells, resulting in progression of carcinogenesis and metastatic disease [38]. When overexpressed, GPx1 has been shown to protect cancer cells from highly oxidizing anti-cancer agents; the level of GPx1 also appears to correlate directly with advanced metastatic cancers [39,40,41].

#### 2.1.2. GPx2

GPx2 is abundantly expressed in the gastrointestinal tract and is also detectible in the human liver. Its substrate selectivity is similar to that of GPx1. The role of GPx2 is considered to be the protection of the GI tract from oxidative damage. In a knockout study of the *Gpx2* gene, increased apoptosis in colon crypt cells was observed during Se-deficiency [42], suggesting that Se-deficiency can affect the GI tract. Research by Emmink et al. indicates that silencing of *Gpx2* results in sensitization to H_2_O_2_-induced apoptosis in patient-derived colonosphere cultures [43]. However, overexpression of GPx2 stimulated tumor growth, as well as differentiation, indicating a redox regulatory function of GPx2 in controlling tumorigenesis.

#### 2.1.3. GPx3

This family member is secreted from the kidney into the blood. Approximately 20% of the Se found in plasma is present in GPx3 [44]. GPx3 appears to serve as an extracellular backup antioxidant for tissues that are exposed to high fluxes of oxidative distress, e.g., the heart [45]. It has been determined that extracellular GSH availability is significantly lower than intracellular [46]. Intracellular available GSH content can vary between 1 and 10 mM, while extracellular GSH content is estimated between 0.002 and 0.8 mM [46,47,48]. The extracellular GSH concentration is highly variable due to tissue location e.g., 0.002 mM in human plasma, up to 0.8 mM in alveolar lining fluid [47,48]. While GPx3 has a lower rate constant, and thus less active than GPx1, it provides antioxidant activity as a peroxidase [49,50]. Therefore, it has been proposed that GPx3, as an antioxidant enzyme, ameliorates potential adverse consequences due to fluxes of ROS. Recycling of GPx3 will take longer due to the relative low availability of extracellular GSH. However, the crystal structure of GPx3 shows thioredoxin folds [50], suggesting a more complicated role for GPx3. Perhaps it has a role in regulating nitric oxide, where repression may lead to cardiovascular disorders [51]. Tumor suppressor functions of GPx3 have also been identified. *Gpx3*-deficient mouse models exhibit increased tumor incidence, as well as altered immune modulation of the tumor microenvironment [52].

#### 2.1.4. GPx4

GPx4, also known as phospholipid hydroperoxide GPx, is associated with membranes, where it can reduce phospholipid hydroperoxides in situ. It has specific isoforms that are ubiquitously expressed for association with cellular membranes. It is localized “on” lipid membranes, in particular the nucleus and mitochondria [53]. GPx4 is a unique GPx family member because it is essential for life. *Gpx4^-/-^* gene knockout murine models were shown to be embryonically (E7.5) lethal [54]. In contrast, heterozygote *Gpx4*^+/-^ mice are viable, but are much more sensitive to lipid peroxidation. However, in the mitochondrial sheet of spermatozoa GPx4 is transformed to function as a structural protein with no enzymatic activity [55].

Interest in GPx4 has increased due to renewed interest in its role in iron-mediated lipid peroxidation that leads to a nonapoptotic cell death, now referred to as ferroptosis [56]. GPx4 regulates lipid peroxidation by chemically reducing phospholipid hydroperoxides to their corresponding alcohol. During ferroptosis, the capacity of GPx4 to remove lipid hydroperoxides is inadequate to deal with the flux of lipid hydroperoxides, leading to cell death [57].

**Figure 1:**
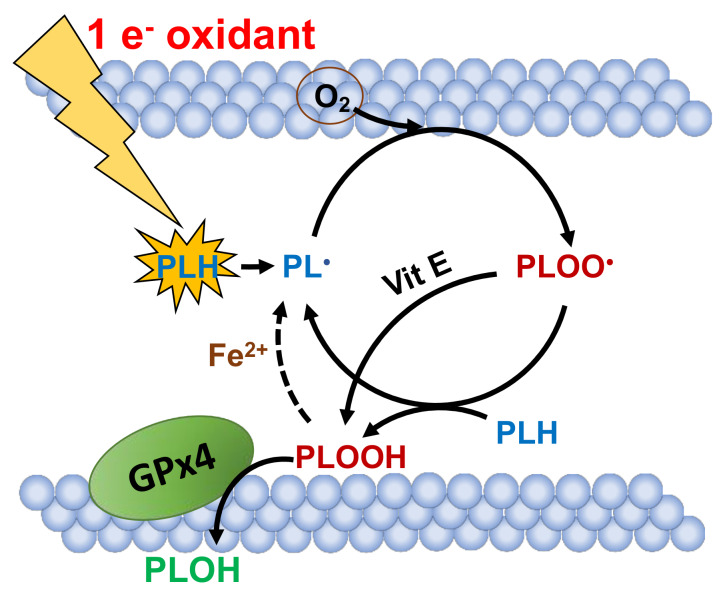
GPx4 activity is central to the termination of cellular lipid peroxidation. GPx4 plays an integral role in preventing iron-mediated lipid peroxidation nonapoptotic cell death, perhaps ferroptosis. GPx4, located in the lipid membrane, inhibits lipid peroxidation (LPO) by removal of PLOOH, a reactive phospholipid hydroperoxide, converting it to a non-reactive alcohol, PLOH. Here, PL^•^ is a carbon-centered radical on a phospholipid chain; PLOO^•^ is a phospholipid peroxyl radical, and PLH represents a phospholipid with the H representing a *bis*-allylic hydrogen that readily reacts with peroxyl radicals to initiate a new chain of lipid peroxidation. Initiation of the first chain is done by a one-electron oxidant. Vitamin E and PLH are competing for reaction with PLOO^•^; sufficient vitamin E will keep the reaction of PLOO^•^ with PLH at a minimum, thereby inhibiting the formation of new chains of peroxidation reactions.

Phospholipid hydroperoxides are formed during propagation reactions of lipid peroxidation. The chain reactions of lipid peroxidation are initiated when a strong one-electron oxidant reacts with a *bis*-allylic hydrogen on a polyunsaturated lipid (Figure 1). This results in the generation of a carbon-centered radical on the phospholipid (PL^•^). This carbon-centered free radical can react with oxygen to form a phospholipid peroxyl radical (PLOO^•^), which can then initiate new chains of free radical-mediated oxidations resulting in the formation of phospholipid hydroperoxides (PLOOH) [58]. Vitamin E can also facilitate the formation of PLOOH. This prevents the formation of a new carbon-centered phospholipid radical, preventing further propagation. If insufficient GPx4 activity is available, the steady-state level of PLOOH increases. Ferrous iron can react through a Fenton-like reaction with PLOOH, initiating new chain-branching reactions [59,60,61]. This in turn, can result in cell death, perhaps ferroptosis.

It is notable that the fundamental basics of this mechanism were observed in the 1950s by Schwarz and Folts [3]. In their studies, liver necrosis in rodents was prevented by Se, while deficient in vitamin E. Their observation supports the collaborative roles of vitamin E and GPx4 in the prevention of chain-branching reactions in lipid peroxidation brought about by redox active iron (Figure 1). However, it appears that GPx4 can compensate when vitamin E is limited. In contrast, if GPx4 activity is deficient, vitamin E alone will not prevent lipid peroxidation in a sustainable manner [62].

Thus, inhibition of GPx4 can lead to increased cell death, making this a potential target for cancer therapies. Currently, the link between GPx4 and ferroptosis is being comprehensively investigated by the scientific community. However, there is still not much unknown about the effects of GPx4 in earlier stages of cancer, e.g., carcinogenesis. In that setting, GPx4 may protect cells and tissues from lipid peroxidation, thereby preventing the initiation of cancer [63,64].

GPx4 can alter the mammalian cell cycle, an important aspect in some cancer therapies [65]. There is also evidence from basic science studies that GPx4 protects cells from the lipid hydroperoxides formed from the generation of singlet oxygen by photosensitizers, a consideration in the mechanism of action of photodynamic therapy [66].

### 2.2. Thioredoxin Reductases

Thioredoxin reductases (TrxR) are homodimeric enzymes with a flavin FAD and NADPH binding site in each monomer [67]. The molecular weight of each monomer is ≈55 kDa. TrxR is present in higher eukaryotes including humans. The primary function of TrxR is reducing the oxidized form of thioredoxin (Trx) (TxrSS) [67,68,69]. The mechanism of this reduction involves FAD, NADPH, two cysteines residues and a selenocysteine near the catalytic site of TrxR. The substrate (TrxSS) oxidizes the Sec and a Cys residue of TrxR forming the reduced form of thioredoxin, Trx(SH)_2_, and a Sec-Cys bond in TrxR. NADPH then provides two electrons to reset TrxR.

**Figure 2:**
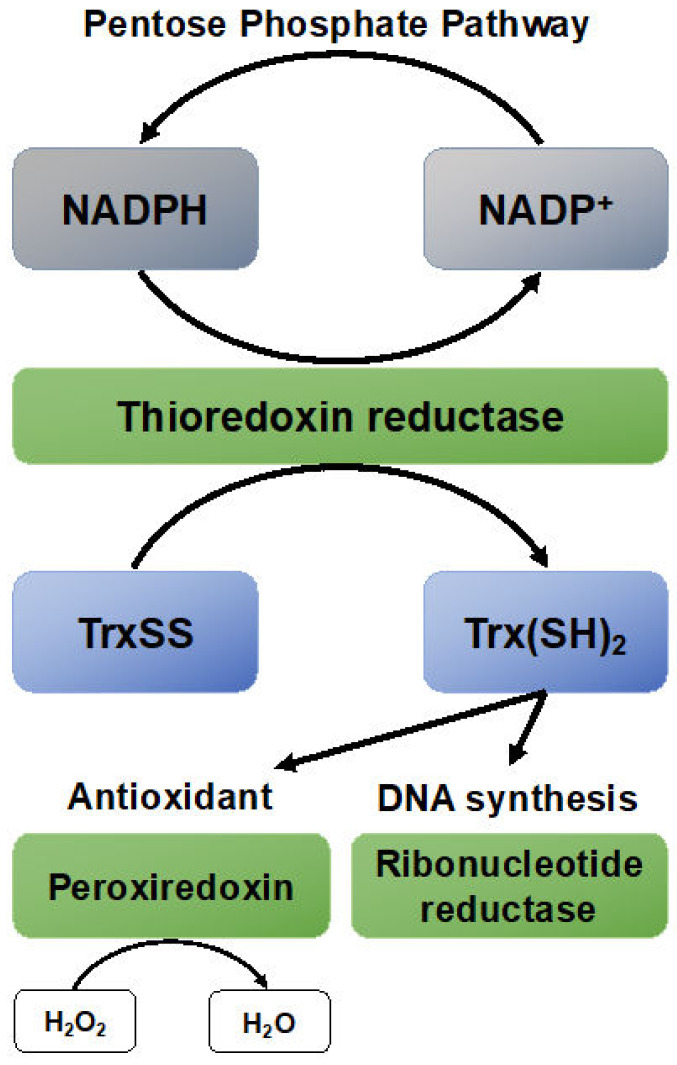
An overview of the function of thioredoxin reductases (TrxR) and thioredoxin (Trx) in cell biology. Trx has multiple roles in its reduced form (Trx(SH)_2_). It can function as a signaling molecule, antioxidant, and is involved in DNA repair and synthesis. Shown are but two of the many functions of Trx(SH)_2_. For example, Trx(SH)_2_ recycles methionine sulfoxide reductase B (MSRB) and is needed for the biology of nitric oxide and hydrogen sulfide.

The major function of TrxR, as the name indicates, is the reduction of thioredoxin. This plays a central role in the thioredoxin node of the redox recycling system [69]. Thioredoxin is utilized for multiple processes. One function is as a secondary antioxidant because it recycles peroxiredoxins after they reduce hydrogen peroxide to water, forming TrxSS (Figure 2) [70]. For the synthesis and repair of DNA, Trx-(SH)_2_ acts as a reducing agent for ribonucleotide reductase, which converts ribonucleotides to 2-deoxyribonucleotides, also known as the D of DNA [71]. Finally, Trx-(SH)_2_ acts as a redox-sensitive signaling molecule that can regulate cell growth, inhibit apoptosis, and it interacts with gene transcription factors like activator protein-1 [72].

Both protein and gene expression of TrxR are affected when selenium is deficient [73,74]. TrxR is up and downregulated when selenium levels are high or in deficiency, respectively. Although, the sensitivity of the individual family members is different, cytosolic Trx (TrxR1) appears to be less sensitive to Se-deficiency, which might be related to the essential nature of the enzyme [75].

In the mid-2000s, thioredoxin reductase was identified as a target for cancer therapy [76,77,78]. Cancer cells typically rely on aerobic glycolysis as a source for energy, known as the Warburg effect [79]. Additionally, a higher amount for glucose is required for the production of reducing equivalents in the pentose phosphate pathway (PPP). In the PPP, glucose is used as a source of electrons to reduce NADP^+^ to NADPH. NADPH is used as a source of electrons that are shuttled down the Trx-cascade to counter oxidative distress (Figure 2). Tumor cells rely on this pathway to maintain redox balance, and thus viability and their capacity for cell division [80,81]. Therefore, inhibition of TrxR can sensitize tumor cells to oxidative damage and enhance tumor-specific cell killing [76,77,82,83]. This makes TrxR a promising target for cancer therapies.

### 2.3. Iodothyronine Deiodinases

The iodothyronine deiodinases (DIO) family, is a family of selenoenzymes with three individual members: types 1, 2, and 3. The enzymes have a molecular weight of 29—33 kDa and share similar sequence homology and catalytic properties [84]. In contrast to many other selenoproteins, DIOs are less involved in oxidative distress processes. DIOs are predominantly active in the thyroid hormonal system, where they convert T4 prohormone to T3. However, DIOs 1 and 3 can also inactive T4 by producing reverse T3, i.e., rT3. The activity of DIO1 is very sensitive to Se-deficiency, similar to GPx1, and can be readily increased by replenishing the Se content of cells and tissues [85]. Whereas, DIOs 2 and 3, like GPx4, reside high in the selenoprotein hierarchy. Selenoproteins high in this hierarchy are prioritized to receive Se when limited [86]. This can be further influenced by non-ubiquitous supply of Se to organs, and sex-specific selectivity. Other factors like iodine deficiency and high levels of thiocyanate in combination with Se-deficiency are related to Kashin–Beck disease or endemic myxedematous cretinism [87]. Several cancer types have been identified that express both high and low levels of DIOs. This can potentially lead to the loss of the control of cell proliferation in these cancers [88]. However, detailed mechanisms and understanding of the contribution of DIOs in cancer are still lacking.

### 2.4. Methionine Sulfoxide Reductases B

Methionine sulfoxide reductase B (MSRB), also known as selenoprotein R (SELENOR) is a Se-dependent family member of the methionine sulfoxide reductases [89]. While other MSR family members have a Cys residue at their active site, MSRB instead has a Sec moiety. The role of MSRB is to reduce oxidized methionine residues (methionine sulfoxide) in proteins back to methionine. Loss of function or knockout of the MSRB gene results in an increase in markers of oxidative distress in liver and kidney tissues, locations where this enzyme is most abundant [90]. In liver tissue this loss may increase the probability for hepatocellular carcinoma [91]. Other possible correlations between MSRB and cancer have yet to be identified.

### 2.5. Selenoproteins

Selenoproteins are not necessarily specified by a function-informing name. Most selenoproteins are as yet understudied and have been identified through genome sequencing [19]. These proteins have a wide variety of functions; some have a similar Cys-X-X-Sec motif like TrxR suggesting redox activity. Other selenoproteins contribute to protein structure via Sec-Cys bonds. A select subset of the better-studied members of this group, such as selenoprotein P (SELENOP), are discussed below. Table 1 provides an overview of most of these proteins with a brief description of their function.

#### 2.5.1. SELENOP

Selenoprotein P is unique in the selenoprotein family. Most Se-containing proteins typically contain one Sec, SELENOP contains up to 10 Sec residues. The large number of Se-containing residues in the protein suggests that it has a role in the transport of selenium. SELENOP knockout mouse models support the role of SELENOP in the transport of dietary Se to tissues [92,93]. SELENOP transports Se specifically into the brain and testes; SELENOP knockout mouse models suffer from neurological problems and male sterility. When dietary intake of Se is limited, liver tissue can readily take up Se to incorporate it into SELENOP, where it is secreted into the plasma for transport to other tissues [94]. Reuptake of SELENOP in tissues is regulated by at least two different receptors: apolipoprotein E receptor-2 for the brain and testis [95,96], and megalin, a lipoprotein receptor in the proximal tubule from the kidney [97]. Since SELENOP is mainly linked to the transport of Se, its expression may be used to determine the prognosis of cancer patients [98]. However, lower SELENOP levels may be caused by insufficient intake of Se and can be simply reversed by supplementation. In addition, SELENOP biosynthesis is decreased during the inflammatory response, i.e., acute-phase response, disrupting Se transport and metabolism of Se [99]. This can potentially contribute to the reduced Se status of cancer patients. Furthermore, a reduction of SELENOP and Se in plasma was observed 1 day after minor elective surgery in mice [100]. These examples clearly show that SELENOP and Se distribution can be blunted by inflammation, with the potential to sensitize humans to oxidative stressors. In addition, single nucleotide polymorphisms in the human *SELENOP* gene show an altered response to dietary intake of Se [101]. This could also limit Se transport, resulting in decreased activities of Se-dependent enzymes and increasing the susceptibility of the initiation of cancer [102].

#### 2.5.2. SELENOF and SELENOM

Selenoprotein M and selenoprotein F are 15 kDa proteins that have 31% similarity in sequence; both are localized in the endoplasmic reticulum (ER) [106]. They contain a typical thioredoxin-like fold and have Cys-X-X-Sec motifs, which suggests redox activity as thiol-disulfide oxidoreductases [105]. Both enzymes are associated with protein-folding in the ER. However, the evidence of this functionality in vivo is scarce [104]. A recent report of SELENOM suggests that it can play a role in the progression of renal cell carcinoma [107]. Furthermore, SELENOF was suggested to play a similar role in colon cancer [108].

### 2.6. Selenium Deficiency in Cell Culture

Many drug development and cancer therapy studies rely on in vitro models to screen for cellular adverse effects. It was suggested in 1985 by Speier et al. that cell culture medium is deficient in selenium [15]. By simply adding selenium to the cell culture medium, maximum GPx activity was achieved. Later, other labs showed increases of selenium-dependent enzyme activities upon supplementation with selenium. Table 2 is a list showing increased enzyme activities in various cell lines once supplemented with Se. These studies indicate that selenium-deficiency prevents maximum Se-dependent activities across cell culture models, thereby impacting single or multiple functions.

## 3. The Role of Selenium in Cancer Prevention

Selenium as a trace nutrient has been proposed to prevent certain types of cancer [113]. In these studies, cohorts were compared that have low vs. high dietary intake of selenium. A systematic review by Vinceti et al. found several studies with inverse associations between selenium intake and the risk of certain types of cancer [10]. However, most studies show no significant cancer preventative properties for Se. The results of a study conducted by the National Cancer Institute, *Selenium and Vitamin E Cancer Prevention Trial* (SELECT), reported an increased risk of prostate cancer development among men supplemented with vitamin E [113,114]. It is important to note that subjects enrolled for this trial had relatively high Se status, 135 ng/mL in serum [115] (normal recommended range ≈ 110–130 ng/mL [116]). In fact, populations in Europe typically have lower Se intake than populations in North America [117]. Therefore, the SELECT study would have been better off with a cohort that was Se-deficient. Such a study may reveal any potential cancer preventative effects of Se in a population that has average intake of Se [115,116].

Various mechanisms have been proposed for how Se might prevent cancer. Several Se compounds have been shown to prevent the initiation of cancer through point mutations that were a result of covalent DNA adducts [118]. Selenium inhibited carcinogenesis by known initiators in mammary, lung, liver, and colon tissues in rats [118,119]. In a later study, selenium-enriched garlic was shown to induce enzymes responsible for phase I and phase II metabolism. This suggested that with Se, carcinogens are more readily detoxified and excreted [120]. On the other hand, induction of phase I metabolic enzymes can bioactivate some carcinogens. For example, members of the cytochrome P450 superfamily can bioactivate polyaromatic hydrocarbons, such as benzo(a)pyrene, to benzo(a)pyrene-7,8-dihydrodiol-9,10-epoxide. This highly reactive metabolic intermediate induces mutations on the tumor suppressor gene p53 [121,122,123]. Hence, it is unlikely that induction of phase I enzymes by selenium-enriched garlic was responsible for the inhibition of carcinogenesis.

Unsurprisingly, most trials have found no significant impact of Se on the incidence of cancer. Clark et al. published a report on a double-blind randomized controlled trial with a cohort of 1312 patients treated with 200 µg × d^−1^ of selenium [124]; the RDA is 55 µg × d^−1^. After a mean treatment period of 4.5 y, no statistical differences were found in the incidence of basal or squamous cell carcinoma of the skin. Results from the secondary endpoints of this study did suggest decreases in the incidence and mortality of other carcinomas. From a phase III clinical trial where Se was tested as an agent to prevent prostate cancer in men with high-grade prostatic intraepithelial neoplasia (SWOG S9917), Marshall et al. (2011) reported a small nonsignificant decrease in prostate cancer risk between patients that were treated with 200 µg daily vs. one of the placebo groups [125]. This subset contained subjects with the lowest quartile of Se concentrations in blood. For all other subsets in this study, Se did not affect prostate cancer risk. In 2013 a Phase III clinical trial (ECOG 5597) reported that 200 µg × d^−1^ did not affect the prevention of the formation of second primary tumors in stage 1 non-small-cell lung cancer patients [126]. In that same year, a Phase III clinical trial conducted at the University of Arizona Cancer Center investigated the effect of Se in men with high risk for prostate cancer; 200 or 400 µg × d^−1^ did not affect the incidence of prostate cancer compared to the placebo-controlled cohort with high risk.

**Figure 3:**
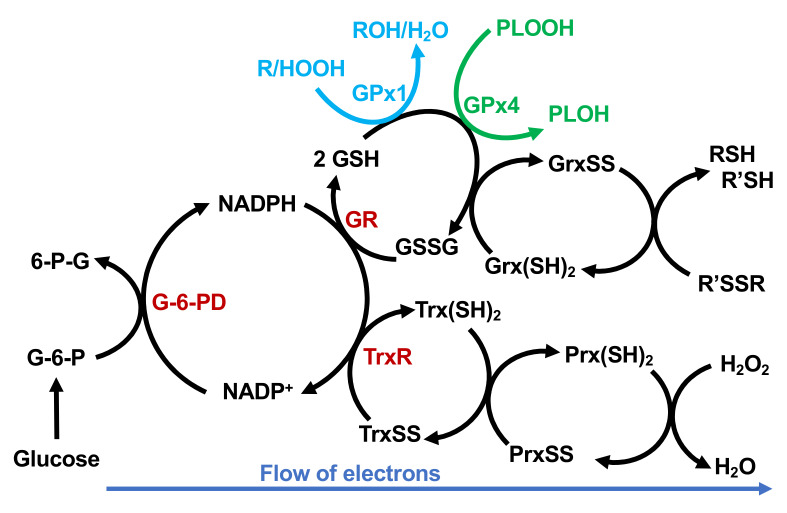
The selenium-dependent antioxidant system of glutathione peroxidases (GPx) and thioredoxin reductases (TrxR). Reducing equivalents necessary to counter unwanted oxidations are obtained from glucose via the pentose phosphate pathway. Glucose-6-phosphate dehydrogenase (G6PD) reduces NADP^+^ to NADPH, which in turn is used by glutathione disulfide reductase (GR) and thioredoxin reductase (TrxR) to reduce glutathione disulfide (GSSG) and oxidized thioredoxin (TrxSS) back to 2GSH and Trx(SH)_2_, respectively. Glutathione “recycles” GPx enzymes, e.g., GPx1 and GPx4. NAPDH is also a cofactor for the reduction of TrxSS, carried out by TrxR. GSH and the GPxs along with Trx(SH)_2_ and peroxiredoxins (Prx(SH)_2_) work via separate nodes to reduce H_2_O_2_ to H_2_O. Adapted from [69].

It is well accepted that optimal selenium intake (≈ 55–75 µg × d^−1^) saturates the expression of selenoproteins in the plasma [7,8,9]. Thus, supranutritional amounts of Se may not provide additional benefit to humans from the selenium-dependent antioxidant system (Figure 3). Other mechanisms have since been proposed to explain the cancer prevention potential of Se. In 1990, Ip and Ganther suggested that redox-active metabolites of selenium may be important in the prevention of cancer [127]. It was later understood that these redox-active metabolites of selenium may not halt the initiation of carcinogenesis, but instead can be used for the treatment of cancer.

Se has also been suggested to protect from DNA damage through redox factor-1 (REF1)-modulated stabilization of p53 [128,129,130]. This oncogene is typically inactive in tumor cells, thus, this effect is thought to protect normal cells during cancer therapies. However, these experiments used cell culture models, and may not have considered that cell culture medium is Se-deficient [18]. Thus, these observations may have been “normal” cell physiology and protection from DNA damage only occurs through this pathway when Se-sufficient.

## 4. Redox Active Selenium

### 4.1. Selenium at an Active Site of an Enzyme Is Typically More Reactive Than Its Sulfur Counterpart

In the periodic table of elements, selenium is located in group 16, i.e., the chalcogens. Thus, it has similar chemical properties in biology when compared to sulfur. A key difference between sulfur and selenium, when incorporated into the active site of enzymes as either Cys or Sec is the p*K*_a_ (Equations (2) and (3)) [131,132]. Sulfur, as thiol, is incorporated into proteins via cysteine (Cys-SH). Selenium, as selenol, is incorporated into proteins and enzymes as selenocysteine (Cys-SeH). At physiological pH = 7.4, the thiol of cysteine remains mainly protonated, ≈ 90% as (Cys-SH). However, the lower p*K*_a_ of selenocysteine results in > 99% being deprotonated, existing as Cys-Se^-^. In general, this difference results in a much greater nucleophilicity of selenocysteine, compared to Cys-SH at pH = 7.4. Thus, Cys-Se^-^ is much more reactive, for example with hydroperoxides. However, it must be kept in mind, that the effective p*K*_a_ of Cys-SH in a protein can vary widely, depending on the local environment.

Cys-SH → Cys-S^−^ + H^+^   p*K*_a_ = 8.5 (2)

Cys-SeH → Cys-Se^−^ + H^+^  p*K*_a_ = 5.2 (3)

Selenium is central to the activity of many redox enzymes (Figure 3). By protecting cells and tissues from oxidative distress, it regulates and maintains the redox status of cells and tissues [133,134,135]. However, inorganic sources for selenium in supranutritional concentrations have been found to exhibit pro-oxidant characteristics [14]. The oxidation state of Se in inorganic sodium selenite and methylseleninic acid is +4; thus, they can directly oxidize cysteine residues in catalytic subunits of enzymes [136]. Many studies on selenium have utilized supranutritional doses of selenium, which show anti-cancer effects. However, when inorganic forms are used, the window for potential toxicity is greatly increased [137]. Thus, it is important to select the appropriate form of Se, which will depend on the goal for its use. The toxic characteristics of inorganic selenium may be preferred.

### 4.2. The Pro-oxidant Side of Selenium

Selenium is generally known for its potential toxicity. In 2009, an overdose of selenium administered intravenously, as selenite, led to the death of 21 polo ponies [138]. This acute toxicosis was proposed to be caused by the oxidation of cysteine residues of proteins and enzymes resulting in their impaired function [13,139]. Human subjects were also exposed to high doses of Se, due to a misformulation of a dietary supplement [140,141]. The estimated mean intake of Se varied between 20,000 and 40,000 µg × d^−1^ (recommend dietary allowance is 55 µg × d^−1^). Thankfully, there were no acute deadly cases, however a large number of short- and long-term adverse effects were observed. Some of the reported symptoms included diarrhea, brittle nails or discoloration, loss of nails or hair, joint pain, and fatigue. In these formulations the source for Se was sodium selenite, an inorganic form of Se. It is comparable to other forms of selenium, e.g., sodium selenate and methylseleninic acid (H_3_CSe(=O)OH), which can be quite toxic due to their ability to oxidize thiols (Equations (4)–(6)), with associated formation of reactive oxygen species such as superoxide and hydrogen peroxide (Equations (7)–(9)) [142,143,144,145,146], while other forms show very little toxicity, for example selenomethionine [18,143,144]. Example reactions with thiols, using glutathione (GSH) as an example thiol, are:

SO_3_^2−^ (selenite) + 4GSH → GSSG + GSSeSG + H_2_O + 2OH^−^ (4)

GSSeSG + GSH → GSSG + GSSeH(5)

GSSeH + xGSH + O_2_ → GSSG + R-SeH + H_2_O_2_ (6)

In Equation (6) GSSeH reacts with additional thiols to form more disulfides as well as H_2_O_2_, potentially through a superoxide intermediate. R-SeH represents an array of Se-containing species. The reactions of methylseleninic acid are parallel:

H_3_CSe(=O)OH + 2GSH → GSSG + H_3_CSeOH (7)

H_3_CSeOH + 2GSH → GSSG + H_3_CSeH (8)

H_3_CSeH + 2GSH + O_2_ → GSSG + H_2_O_2_ (9)

In Equation (9), H_3_CSeH (methyl selenol) reacts with additional thiols, probably via H_3_CSe^−^ (p*K*_a_ (H_3_CSeH) = 5.2), catalytically generating H_2_O_2_ and additional disulfides [143]. Although the reactions above are shown with GSH, these reactions would also occur with other cellular thiols including protein thiols. Thus, not only will GSH be depleted, but also NADPH. The oxidation of thiols in proteins to disulfides, including mixed disulfides, will have many downstream consequences, including enzyme inactivation.

As an example, Se can decrease the DNA binding affinity of NFκB and AP-1 by facilitating the oxidation of cysteine residues, resulting in a reduction of cytokine production [147,148,149]. Other redox-sensitive enzymes, as well as redox signaling proteins are shown to be affected by high levels of selenium. Thiol oxidation, induced by selenium, inhibited activities of several kinases, caspase-3, prostaglandin synthesis, and mitochondrial function [148,150,151,152,153,154,155,156]. These examples clearly show the wide range of targets that could be altered by selenium-induced oxidation.

### 4.3. Cancer Therapies Utilizing Selenium

The potential to increase cellular oxidative distress with appropriate Se-compounds could be an important consideration in cancer therapy. Redox-active Se-compounds have pro-oxidant properties, increasing the formation of ROS. Cancer cells are thought to have higher steady-state levels of ROS compared to their normal cell counterparts [133,157,158,159,160]. Therefore, combining these properties may tip the oxidative balance of cancer cells to an even greater degree of distress by generating additional reactive oxygen species, e.g., superoxide (O_2_^•-^) and hydrogen peroxide (H_2_O_2_). In general, normal cells can manage an increase in the flux of oxidants. However, it is proposed that cancer cells are at their limit of ability to control oxidative distress. Generating more ROS through an appropriate pro-oxidant Se-compound would take advantage of these differences, providing a therapeutic advantage.

Many publications show the pro-oxidant side of selenium compounds. Sodium selenite and selenocystamine have been shown to increase oxidative DNA lesions, e.g., 8-hydroxydeoxyguanosine [142]. Additionally, apoptosis appears to be readily induced by sodium selenite, as well as single-strand DNA breaks [161,162,163,164,165]. However, selenomethionine does not exhibit similar adverse effects.

Inhibition of neoplastic growth was shown by redox-active selenium, selenite (5 µM), selenium-di-glutathione (5 µM) in HeLa Cells. However, a non-redox active selenium compound, Se-DL-cystine showed inhibition at much higher concentrations (100 µM) [166]. Furthermore, changes in the half-cell reduction potential (more positive) were observed as more GSH was oxidized. These observations suggest that these compounds cause cell death to HeLa cells via three different pathways. Overall, selenite led to a more positive redox environment in cells resulting in necroptosis.

Selenium-di-glutathione was suggested to cause glutathionylation/oxidation of death receptors resulting in apoptosis. Whereas, Se-DL-cystine causes ER stress, resulting in apoptosis or cell death [166].

Due to the ROS formed and the ability to oxidize intracellular thiols, redox-active selenium compounds can activate Nrf2 [167]. Upon oxidation of Keap1, Nrf2 is released in the cytosolic fraction of cells [168]. Nrf2 then acts as a transcription factor binding to the antioxidant response element (ARE) [169]. Activation of the ARE upregulates the transcription of a large array of antioxidant, phase I, and phase II detoxifying enzymes [170,171]. Two of these target enzymes are selenoenzymes: GPx2 and TrxR1 [172]. It is now understood that activation of Nrf2 can protect normal cells against the initiation of cancer. However, transformed cells will most likely benefit equally from this phenomenon, resulting in further progression of the disease [167].

In addition to small molecular forms of Se, nanoparticles of Se have been proposed for use in cancer treatment. The main benefit of nanoparticles in cancer treatment is added functionality. A monolayer of molecules functionalizes the particles to gain specific properties. Liu et al. functionalized Se nanoparticles (300–500 nm) with folate [173]. This guides the nanoparticles to cancer cells, which often overexpress the folate receptor. The core of the nanoparticles was made of selenite, which is released locally, upon binding to the folate receptor. This specific model showed relatively high cytotoxicity in HepG2 and multi-drug resistance HepG2 cell lines. In contrast, there was no change in hematological values, nor was liver toxicity observed in female rats exposed to a relatively high dose of 5 mg kg^-1^ Se nanoparticles (20 nm) [174].

Selenium can also be used in supplemental quantities, < 0.5 mg d^-1^, as an adjuvant for chemo and radiation therapy [175,176]. Muecke and colleagues suggested that cancer patients that have been treated with chemo or radiation therapy could benefit from supplementation of Se. It could restore selenium-deficiency and alleviate side-effects of cancer therapies. Se-deficient patients were enrolled for a phase 3 clinical trial, and Se successfully reduced radiation therapy induced diarrhea, while not affecting the effectiveness of the anticancer therapy.

In 2014, the 10-year survival rate seen in this phase 3 trial was increased in the Se supplemented group, albeit not significant, *p* = 0.09 [175]. It is interesting whether this effect was caused by restoring the selenium-dependent antioxidants due to possible Se-deficiency or other less familiar mechanisms. At these low, supplemental concentrations, Se may only have a restoring effect for selenoproteins and enzymes, since most selenium compounds do not start to show any toxicity at these lower concentrations.

Selenium compounds can have a large variety of properties (Table 3). It is important to understand which compound to select for use in an experimental design. Selenium compounds have been developed to have slightly different properties; however, one major property is shared; any selenium compound appears to increase selenoenzyme expression and activities when the amount of accessible Se is limited [133]. For example, supplementing Se-deficient cell culture media with 500 nM SLM or sodium selenite, will both achieve maximum GPx1 activity. Yet, sodium selenite can increase the flux of superoxide at 1–10 µM concentrations, whereas SLM will not. Therefore, it is important to understand the different properties of Se compounds during the design of any biomedical experiments. In cancer therapy, inorganic forms, such as sodium selenite, are most likely preferred due to their pro-oxidant nature.

### 4.4. High Dose Organic Selenium

Most cancer therapies that utilize selenium rely on the pro-oxidant properties of inorganic Se. However, at The University of Iowa seleno-L-methionine (SLM), an organic source for Se, is being used in a clinical trial (NCT02535533) [177,178]. This phase I/II trial employs high dose SLM as an adjuvant in the treatment of renal cell carcinoma (RCC). SLM can be administered in much higher doses compared to inorganic forms [137]. In contrast, the therapeutic window of inorganic forms of Se is much smaller due to dose-limiting toxicities [179]. At these high doses of SLM, new therapeutic phenomena occur. SLM, as well as methyl-selenocysteine, show enhanced drug delivery to the tumor [180,181,182]. In addition, these compounds have been shown to effectively inhibit Hypoxia-Induced Factors-1α and -2α (HIFs), which are highly expressed in RCC [183,184,185,186,187]. SLM is currently being tested in metastatic RCC patients in combination with Axitinib, a vascular endothelial growth factor receptor (VEGFR) inhibitor in recurrence and refractory setting. SLM at different dose levels (2500, 3000, and 4000 µg) was administered orally twice daily for a 14-d run-in period, followed by once daily dosing in combination with Axitinib 5 mg twice daily until disease progression or unacceptable toxicity. Eligible patients must have clear cell RCC histology and have progressed on at least one prior treatment. The primary objective of the study is to evaluate the safety of the combination, and the recommended Phase 2 dose (RP2D) of SLM, secondary endpoint is to evaluate the preliminary efficacy as measured by objective response rate (ORR), progression free survival (PFS) and overall survival (OS). Preliminary results of 12 evaluable patients were presented at ESMO 2019 [188], showing that high dose SLM, 4000 µg twice daily for 14 days followed by once daily, in combination with Axitinib, was safe with promising efficacy; two patients achieved complete response (CR) for at least 2 years; four patients achieved partial response. No dose-limiting toxicities were noted. A Phase 2 trial is currently ongoing with SLM 4000 µg and Axitinib 5 mg twice daily. This research has opened new opportunities to use Se as a drug, rather than a supplement.

SLM can be incorporated into proteins and enzymes instead of a methionine [189,190]. This may lead to difficulties to interpret the information gained from a clinical trial. Besides changes in Se-dependent proteins and enzymes, many other factors change, which makes it hard to pin-point what caused the effect [116].

## 5. Discussion

Selenium-dependent enzymes have many roles in the maintenance of optimal human health. The majority of selenoproteins and enzymes have roles in the prevention of carcinogenesis. However, selenium deficiency decreases the expression and activities of selenoproteins and enzymes, affecting their cancer preventative properties. Thus, it is important to prevent selenium deficiency.

Cell culture studies are essential basic studies for many labs around the world. However, selenium deficiency in the media is often not considered by laboratories that use cell culture models. Cell culture medium, containing 10% fetal bovine serum as a sole source for Se, is typically quite deficient in Se. This reduces the antioxidant capacity of cells; these cells are less able to handle an oxidative challenge. We have proposed that supplementation with Se should always be an integral part of any cell culture experiment [18].

Selenium has long been known for its toxicity. However, the toxicity of Se is determined by its molecular structure. Organic forms of selenium are typically better tolerated by humans in higher quantities. These organic forms are also the predominant form in our daily intake, typically as selenomethionine. Inorganic sources are taken up from the soil by plants and converted to organic forms e.g., selenomethionine. Adequate availability of Se maximizes the expression and activities of Se-dependent proteins and enzymes. Many of these play an important role in the antioxidant system. This system is thought to protect cells and tissues against oxidants that are formed from cellular processes as well as xenobiotic metabolism. It is therefore hypothesized that Se supplementation can protect against the initiation of cancer. However, conclusive evidence of a correlation between high Se intake and decreased cancer incidence is still lacking. It is more likely that many populations are adequate in selenium with a balanced diet and multivitamin supplements (including Se). Higher than adequate intake of Se will not further increase Se-dependent antioxidant enzymes. Therefore, it is unlikely that high Se intake can protect against the initiation of cancer through this mechanism. Populations that suffer from Se deficiency may benefit from Se supplementation for the prevention of cancer. It has been estimated that 25% of the world’s population is sub-clinically Se-deficient [202]. Meaning that ≈ 25% of the population is Se-deficient without clinically observed adverse effects. In this sub-population, maximum Se-dependent antioxidant activities will not be achieved, suggesting that this group may have an increased risk for the development of health issues, including cancer [113] as well as cardiovascular disease [203,204]. Potentially, this could be prevented by supplementation with Se.

Redox-active selenium compounds, in supranutritional doses, can form reactive oxygen species (ROS). Selenium-dependent oxidation of thiols can directly result in the formation of superoxide. This will be dismuted by superoxide dismutases, forming hydrogen peroxide, which in the presence of ferrous iron generates hydroxyl radical. These ROS contribute to the damage of essential subcellular organelles, thereby driving apoptosis and necrosis in cells and tissues. These properties of Se are also suggested to prevent further progression of cancer. Transformed cells have a higher steady-state level of ROS compared to their normal cell equivalent. Therefore, the addition of redox-active Se can damage cancer cells, because cancer cells lack sufficient reducing capacity to detoxify the oxidants formed [133,157,158,159,160]. Normal cells have a relatively greater reducing capacity and will survive the challenge of redox-active Se. In addition to redox-active forms of Se, organic selenium, e.g., seleno-L-methionine (SLM), is also used for cancer treatment. It has been proposed that organic forms of Se change the vascularity of the tumor resulting in better drug delivery. The use of high doses of SLM as an adjuvant to chemotherapy may greatly improve the outcomes. Furthermore, the use of Se as an adjuvant to chemotherapy and radiation therapy has been shown to reduce the side-effects while not impacting the main oncological effects of chemo and radiation therapy [176,205].

## Figures and Tables

**Table 1:** An overview of 24 selenoproteins and some of their functions.

Selenoprotein	Function	Reference
GPx1	Reduction of solubilized organic hydroperoxides and hydrogen peroxide in the water space of cells, cytosol, mitochondria, nucleus	[23,103]
GPx2	Abundantly expressed in tissues of the liver and GI tract. Similar function to GPx1	[42]
GPx3	Extracellular GPx, regulation of nitric oxide	[45,51]
GPx4	Reduction of lipid hydroperoxides, essential for the termination of lipid peroxidation; prevents ferroptosis	[53,73]
GPx6	Only found in humans, no specific role has yet been identified	[19]
TrxR1	Reduces TrxSS to Trx(SH)_2_, vitamin C, polyphenols, and other substrates to regulate intracellular redox environment	[67]
TrxR2	Located in mitochondria, control and regulates redox environment	[19]
TrxR3	Abundant in testes, reduces mitochondrial glutathione disulfide	[19]
DIO1	Converts T4 (thyroxine) into T3 (active thyroid hormone) and inactivates T4 to rT3	[84]
DIO2	Converts T4 into T3	[84]
DIO3	Inactivates T4 to rT3, as well as T3 to T2	[84]
MSRB	Restores oxidatively damaged methionine (Met-sulfoxide) to native configurations	[89]
SELENOF	Oxidoreductase that may assist in disulfide formation and protein folding	[104]
SELENOH	Regulates GSH synthesis during embryonic development	[19]
SELENOI	Functions as a phospholipid synthase	[19]
SELENOK	Abundant in myocytes, function is currently unclear	[19]
SELENOM	Abundant in myocytes, function is currently unclear	[105]
SELENON	Interacts with ryanodine receptor, mutations result in congenital muscular dystrophy	[73]
SELENOO	Suggestive redox function due to Cys-X-X-Sec motif	[19]
SELENOP	Plasma selenium transport protein, contains up to 10 Sec, exhibits very low GPx4-like activity when purified	[92,93]
SELENOS	Suggested to be involved in ER stress response	[19]
SELENOT	Suggested role in Ca^2+^ mobilization	[19]
SELENOV	Suggestive redox function due to Cys-X-X-Sec motif	[19]
SELENOW	Expressed in a variety of tissues and may regulate redox state of 14-3-3 proteins	[19]

**Table 2:** Supplementing cell culture media with Se increases Se-dependent enzyme activities.

Concentration Supplemented ^a^	Enzyme Studied	Fold Increase ^b^	Cell Line	Reference
4 nM	GPx1	3	Human bronchial epithelial	[109]
50 nM	GPx1, GPx4	3–15 ^c^, 3–10 ^c^	L929, HepG2, D10N, THP-1, ECV 304	[17]
100 nM	GPx1, TrxR	2, 1.5	Bone marrow stromal	[110]
100 nM	GPx1, GPx4, TrxR	2, 2, 1.5	Jurkat, T-leukemia	[111]
200 nM ^d^	GPx1, GPx4, TrxR	3, 3, 2	HepG2, MIA PaCa-2	[18]
2 µM	GPx1	17	RAW 264.7 macrophage	[112]

^a^ as sodium selenite, ^b^ fold increases vs. selenium deficient, ^c^ fold increase range over cell lines, ^d^ as selenomethionine.

**Table 3:** Selenium compounds and corresponding properties.

Compound	IC_50_^a^ (µM)	Comments	Reference
Sodium selenite	1–10	Inorganic source for Se Most widely used Se source Pro-oxidant in high concentrations Administered IV to reach pharmacological doses	[142,166]
Sodium selenate	1–10	Inorganic source for Se Suggested to play a role in obesity	[191]
Seleno-L-methionine	>100	Organic source for Se Abundant dietary source for Se Can be incorporated in proteins as Se-methionine Relative low toxicity	[137,142,192]
Methyl selenocysteine	>100	Organic source for Se Inhibitor of angiogenesis	[133]
Methyl seleninic acid	1–10	Synthetic organic Se compound Potential to alter the immune response in tumors Pro-oxidant properties Enhances tumor cell killing	[193,194]
Selenium-di-glutathione	5	Organic source for Se Product of selenite reduction by GSH	[166,195]
Se-DL-cystine	5–100	Organic source for Se Both antioxidant and pro-oxidant Exhibit GPx-like activity under reducing conditions	[133,166]
Selenocystamine	25–50	Organic source for Se Non-catalytic Prevents DNA oxidation	[142]
Se-adenosyl-L-selenomethionine	N.D.	Synthetic organic Se compound Proposed substrate for methyltransferases	[196]
Ebselen	5–50	Synthetic organic Se compound GPx mimic Antioxidant scavenger Anti-inflammatory	[197,198,199]
Selenophene	0.1–10^b^	Synthetic organic Se compound Antioxidant properties Protect against CCl_3_ induced liver damage Molecular building block for anti-cancer agents	[200,201]
Selenium nanoparticles	<1	Synthetic inorganic Se particles Much lower apparent toxicity in vivo Induces oxidative stress in vitro	[173,174]

^a^ IC_50_ as inhibition of growth, determined in vitro. ^b^ Selenophene is considered a “building block” molecule. Adding functional groups to the main ring structure can change the toxicity of selenophene, making it highly variable and customizable.
